# Current Antithrombotic Therapy Strategies in Children with a Focus on Off-Label Direct Oral Anticoagulants—A Narrative Review

**DOI:** 10.3390/children9071093

**Published:** 2022-07-21

**Authors:** Stefana Maria Moisa, Laura Mihaela Trandafir, Crischentian Brinza, Ingrith Crenguta Miron, Elena Tarca, Lacramioara Ionela Butnariu, Alexandru Burlacu

**Affiliations:** 1 Pediatrics Department, University of Medicine and Pharmacy “Grigore T. Popa”, 700115 Iasi, Romania; stefana-maria.moisa@umfiasi.ro (S.M.M.); laura.trandafir@umfiasi.ro (L.M.T.); ingrith.miron@umfiasi.ro (I.C.M.); ionela.butnariu@umfiasi.ro (L.I.B.); 2 “Sfanta Maria” Clinical Emergency Hospital, 700309 Iasi, Romania; 3 Faculty of Medicine, University of Medicine and Pharmacy “Grigore T Popa”, 700115 Iasi, Romania; alexandru.burlacu@umfiasi.ro; 4 Institute of Cardiovascular Diseases “Prof. Dr. George I.M. Georgescu”, 700503 Iasi, Romania

**Keywords:** thrombosis, pediatric, direct oral anticoagulants, guidelines, off-label

## Abstract

(1) Background: The incidence of thromboembolic events is relatively low in the general population, but it increases in hospitalized children and those who underwent thrombogenic procedures. Although the evidence regarding direct oral anticoagulants (DOACs) in children with venous thromboembolism (VTE) is growing, DOACs were excluded from existing guidelines due to the lack of reliable data at that moment. Therefore, current evidence on VTE management in children needs to be critically reviewed. (2) Methods: We have conducted a literature search in the Scopus, EMBASE, and MEDLINE databases using prespecified keywords to retrieve studies published between 2010 and 2022. (3) Results: Clinical trials highlighted that rivaroxaban and dabigatran had predictable pharmacokinetic and pharmacodynamic profiles in children, similar to those observed in adults. Dabigatran and rivaroxaban had a similar safety profile to standard therapy but improved thrombotic burden and resolution during follow-up. Most studies involving apixaban and edoxaban are ongoing, and results are awaited. (4) Conclusions: Dabigatran and rivaroxaban could be valid therapeutic options for VTE management in children. In the case of apixaban and edoxaban, results from ongoing clinical studies are required before using them in pediatric VTE.

## 1. Introduction

Thrombosis occurs due to an imbalance between procoagulant, anticoagulant, and fibrinolytic systems activation. The reported incidence of thrombotic events in children varied in clinical studies, ranging between 0.07–0.14/10,000 in all patients and 58/10,000 in pediatric patients who underwent potentially thrombogenic procedures [1].

Two incidence peaks were recorded in children, with a higher thrombotic burden in the infant and the adolescence periods. In newborns, the most frequent thrombotic events included renal or cava veins thrombosis, stroke, and pulmonary embolism. The latter could also be observed in older children, along with cerebral or portal venous thrombosis and mesenteric thrombosis [1,2].

The standard treatment recommendations in pediatric patients with venous thromboembolism (VTE) include low-molecular-weight heparin (LMWH) or unfractionated heparin (UFH), which requires activated partial thromboplastin time (aPTT) or activated coagulation time (ACT) monitoring [3,4]. In selected cases, thrombolysis or thrombectomy can also be considered, followed by oral anticoagulation therapy (OAT). In addition, OAT duration varies with clinical and biological contexts. OAT strategy has improved over the last years once direct oral anticoagulants (DOACs) were introduced in clinical practice, encompassing extensive clinical research data, including in the pediatric population [3,4].

Direct thrombin inhibitors (dabigatran) and factor Xa inhibitors (rivaroxaban, apixaban, and edoxaban) constitute an intriguing alternative to traditional anticoagulant therapies. Bodyweight-adjusted rivaroxaban doses were tested in a randomized, controlled, phase III study [5]. Additionally, the Food and Drug Administration (FDA) approved rivaroxaban for venous thromboembolism treatment and for children with congenital heart disease who underwent Fontan surgery [6]. In addition to rivaroxaban, the FDA and the European Medicines Agency (EMA) released recommendations on dabigatran pediatric use for VTE treatment [7,8].

Nevertheless, DOACs were excluded from existing guidelines and recommendations due to the lack of reliable data at that moment [3,4]. Therefore, current evidence on thrombotic events management in children needs to be critically reviewed. This paper aims to appraise available data regarding antithrombotic therapy in the pediatric population, focusing on DOACs’ opportunities and challenges in specific clinical contexts.

## 2. Materials and Methods

We have conducted a literature search in the Scopus, EMBASE, and MEDLINE databases using the following keywords: dabigatran, rivaroxaban, apixaban, edoxaban, children, pediatric, novel anticoagulants, and DOACs. We sought to retrieve studies published between 2010 and 2022; the last search in databases was performed on 10 March 2022.

Studies were considered to be eligible if they met several inclusion criteria grouped in the PICO framework: P—patients aged ≤ 18 years, diagnosed with VTE; I—DOACs (dabigatran, apixaban, rivaroxaban, edoxaban) therapy in all evolution phases of VTE, including for secondary prevention; C—standard anticoagulant therapy, represented by heparin therapy, VKAs, or no therapy (in case of studies with a comparator arm); and O—safety (major or clinically relevant nonmajor bleeding events) and efficacy outcomes (recurrent VTE, thrombosis resolution, or death). Studies that did not report original data regarding DOACs use in pediatric patients, editorials, and those available only in the abstract were excluded from qualitative analysis.

The search was conducted without additional filters such as language restrictions or study design (observational studies and randomized clinical trials were screened for eligibility). Only published studies were included in the qualitative analysis. Nevertheless, we also searched the ClinicalTrials.gov database to identify ongoing studies and to recognize current research directions and expectations.

Following the databases search, two of the independent reviewers screened obtained records for eligibility in a two-step technique. In the first step, titles and/or abstracts were critically appraised; if the inclusion criteria were met, studies were evaluated in full text in the second step. In addition, duplicate studies were excluded using an automation tool (EndNote). Any disagreements were solved by discussion and consensus.

Ten articles were compiled to draw relevant conclusions.

## 3. Summary—Findings from Clinical Trials

Findings from relevant studies were structured to provide a clear overview of VTE management in children, including pathophysiological and epidemiological data, traditional antithrombotic therapies advocated by the guidelines, and the advantages and drawbacks of DOACs therapy.

### 3.1. Thrombosis Pathways

Three major risk factors for thrombosis were described previously by Virchow: blood flow abnormalities, vascular wall abnormalities, and the disparity between procoagulant and anticoagulant factors activity. However, the involvement of the three classical factors might vary in arterial versus venous thrombosis. In this regard, endothelial lesions and platelet dysfunction are critical regulators in arterial thrombosis, while venous thrombosis implies stasis and abnormal function of procoagulant and fibrinolytic systems [9].

Some factors are considered to be protective against VTE in children. These factors include physiological deficiency of specific coagulation factors leading to decreased thrombin-generation ability, enhanced capability of α2-macroglobulin to inhibit thrombin, the high antithrombotic potential of the vascular wall, and the lack of exposure to acquired thrombotic factors such as smoking and antiphospholipid antibodies [10].

### 3.2. Thrombotic Events in Children: Numbers and Outcomes

Thrombosis incidence was higher in children during the first year of life and adolescence [1]. Although thrombosis incidence was relatively low in the general population (0.07–0.14/10,000), it increased in children admitted to the hospital (5.3/10,000) and in those who underwent potentially thrombogenic procedures (58/10,000) [1].

Hospitalized children had a higher incidence of VTE (42–58/10,000), attributed to the following risk factors: venous catheterization, perinatal asphyxia, hypovolemia, malignancy, sepsis, congenital heart malformations, trauma, surgical interventions, hereditary or acquired thrombophilia, hormone therapy, systemic lupus erythematosus, nephrotic syndrome, varicella, the Fontan procedure, and genetic mutations (factor V Leiden mutation, antithrombin III, protein C, and protein S-deficit) [11].

Even though thrombotic events were recorded more frequently in adult patients, morbidity and mortality rates in children were still high. As documented in a report from a Canadian registry involving children with thrombophilia, pediatric patients exhibited an 8.1% thrombosis recurrence rate, 12.4% post-thrombotic syndrome rate, and 2.2% VTE mortality risk during long-term follow-up [12].

### 3.3. Classical Antithrombotic Approach

The mainstay of antithrombotic treatment in children constitutes LMWH and UFH. UFH is preferred in children with renal impairment and those with a high bleeding risk due to its reduced halftime, while LMWH is associated with lower thrombocytopenia risk. Heparin therapy is monitored using aPTT or ACT in case of UFH or anti-Xa factor activity for LMWH. LMWH should be titrated to maintain anti-Xa factor activity between 0.5 and 1.0 units/mL at 4–6 h after administration [13,14].

It is reasonable to continue heparin therapy for at least five days during the acute phase of a thrombotic episode (deep vein thrombosis—DVT, pulmonary embolism—PE). Afterward, vitamin K antagonists (VKAs) can be used as an alternative to heparin during the subacute phase. Following the first thrombotic episode, anticoagulant therapy should be extended up to 3 months or 6–12 months for unprovoked VTE [13].

### 3.4. DOACs in Children—The Future of OAT?

DOACs might constitute a feasible alternative for VKAs in children and have already been successfully and extensively used in adult patients. The interest in DOAC therapy in children emerged due to a more predictable effect than VKAs, with similar or better safety and efficacy profiles [15]. They interact less with other medications, have fewer food restrictions, and have more predictable pharmacokinetics that allows for fixed dosage based on renal function and a reduced need for monitoring [16,17].

DOACs have a different mechanism of action as compared to traditional anticoagulants. While VKAs inhibit all vitamin K-dependent coagulation factors (II, VII, IX, and X), UFH and LMWH activate antithrombin, which inhibits factors II, IX, X, and XI. Distinctively, DOACs act by directly inhibiting thrombin or factor Xa, preventing fibrinogen conversion into fibrin [18]. Therefore, DOACs were tested in some trials involving pediatric patients, thus establishing a background for future integration in guidelines (Table 1).

#### 3.4.1. Dabigatran—The Flagship of DOAC in Children

Dabigatran was the first oral DOAC approved by FDA to treat VTE in children aged > 3 months following heparin therapy for at least 5 days [7,28]. The FDA recommendation was based on the results from several clinical studies.

Pharmacokinetics and pharmacodynamics in pediatric patients with VTE were tested in a nonrandomized, open-label, phase IIa study [20]. Eight full-term neonates and infants aged < 1 year treated with UFH or LMWH were enrolled in the study. Administration of a single dose of dabigatran suspension-adjusted by the bodyweight or renal function exhibited similar concentration profiles as in adult VTE patients. Notably, none of the patients experienced bleeding events, death, or other adverse events linked to dabigatran during the follow-up period of 37 days [20].

Dabigatran was investigated in another phase IIa study involving 18 children aged 1–12 years who completed standard anticoagulant treatment (UFH, LMWH, or VKA) for VTE. This study administered dabigatran for at least five days (equivalent to an adult dose of 150 mg twice daily) compared to the previous study, which used a single dose of dabigatran. The results were concordant, with no reported deaths, bleeding events, recurrent VTE, or other severe adverse events attributed to dabigatran [19]. These data established the background for more extensive clinical studies.

One of the pivotal clinical studies to evaluate the efficacy and safety of dabigatran in children with VTE was the DIVERSITY trial, a randomized, open-label, phase IIb/3 study [21]. A bodyweight- and age-adjusted dabigatran dose was compared to standard anticoagulant regimens (UFH, VKA, fondaparinux) in 328 patients aged < 18 years. Participants were enrolled if they underwent parenteral anticoagulant treatment for 5–21 days before randomization. The primary composite efficacy outcome (complete thrombus resolution, freedom from recurrent VTE, and death linked to VTE) was similar between the treatment groups (46% in dabigatran patients and 42% in the comparator group). The bleeding events rate tended to be slightly lower in the dabigatran group, though it was not statistically significant (22% versus 24%; HR 1.15, 95% CI, 0.68–1.94, *p* = 0.61). A similar major bleeding events incidence was reported in both groups (2%; HR 0.94, 95% CI, 0.17–5.16, *p* = 0.95). Hence, dabigatran could be a treatment option for children presenting with VTE [21].

Dabigatran proved effective in secondary prevention of VTE in children treated with standard antithrombotic therapy for at least three months, as well as in those from the DIVERSITY trial who completed dabigatran or classic anticoagulant regimens [22]. Children aged between 3 months and 18 years (n = 203) were enrolled and followed-up with for 12 months. Recurrent VTE was documented in 1.0% of patients, while 1.5% of participants had a major bleeding event. The authors did not report any deaths related to VTE or dabigatran therapy. Consequently, dabigatran might be considered for long-term secondary prevention in children requiring anticoagulation following a VTE event [22].

The population enrolled in the DIVERSITY trial could be regarded as the reference to guide the clinical decision of dabigatran initiation in pediatric patients with VTE [21,29]. Dabigatran should be avoided in patients with pre-existing conditions associated with an increased risk of bleeding, renal dysfunction (estimated glomerular filtration rate—eGFR < 50 mL/min/1.73 m^2^ or requiring dialysis), hepatic impairment (active liver disease, alanine aminotransferase, or aspartate aminotransferase > 3 × upper limit of normal—ULN), active endocarditis, heart valve prosthesis, and in children aged < 2 years with low body weight (lower than the third percentile) or whose gestational age at birth was <37 weeks. These patients were excluded from the DIVERSITY trial; therefore, there are no available data to support dabigatran administration in the subset mentioned above of children [21,29].

#### 3.4.2. Rivaroxaban—Evidence from Clinical Studies

Rivaroxaban is administered by oral route [30]. Two-thirds are metabolized by cytochrome P450, and the kidney eliminates one-third. Rivaroxaban has a halftime of 5 to 9 h [31]. Rivaroxaban was initially evaluated by EINSTEIN-Jr phase I studies that enrolled children aged ≥ 6 months with prior-treated VTE [23,24]. These studies recorded a predictable effect of a bodyweight-adjusted dose of rivaroxaban in all age subgroups of children (6 months–18 years), providing the context for further phase II and III studies [23,24].

Three multicenter phase II studies were conducted as a part of the Einstein-Jr study program that ended in June 2019 [25]. These studies established the optimum dose regimen for newborns up to 18. In this regard, 93 patients were enrolled with previously treated VTE or catheter-related VTE, including children younger than 6 months. Rivaroxaban was administered via tablets or suspension 1–3 times a day, according to age and weight category. Only four children had a nonmajor bleeding event, while none of the patients experienced major bleeding. The authors did not report any VTE recurrences concerning efficacy endpoints, while 32% of the children had no imagistic evidence of thrombosis during follow-up [25].

Following optimistic results from phase II studies, a phase III randomized controlled clinical trial was conducted [5]. Many patients (n = 500) aged 0–17 years with acute VTE treated initially with heparin were enrolled in multiple centers. Patients were randomized to receive an adjusted rivaroxaban dose or traditional antithrombotic therapy (heparin or VKAs). Even though the symptomatic VTE recurrence rate tended to be lower in the rivaroxaban group compared to patients treated with conventional anticoagulants (1% and 3%, respectively), it was not statistically significant (HR 0.40, 95% CI, 0.11–1.41). Interestingly, complete thrombus resolution during follow-up was documented in 38% of patients treated with rivaroxaban and 26% of control patients (*p* = 0.012). The risk of major or clinically relevant nonmajor bleedings was similar across different anticoagulation strategies (HR 1.58, 95% 0.51–6.27). Notably, all reported bleeding events in the rivaroxaban group were nonmajor, while two patients who received heparin or VKAs experienced major bleeding events. This study highlighted that the adjusted rivaroxaban dose was noninferior to standard anticoagulants in efficacy and safety outcomes, even in young children, but with an improved thrombosis resolution rate [5]. Similar results were found in another phase III trial involving 335 children, with rivaroxaban being a standing alternative to standard anticoagulant therapies in VTE [26].

Concerning rivaroxaban administration and dosing strategies, in phase III trials, children received film-coated tablets or granules used to create a suspension for oral use with a concentration of 1 mg/1 mL. In addition, the rivaroxaban dose varied across bodyweight subgroups, ranging from 2.7 mg daily (three times daily) in those <4 kg to 20 mg once daily in those >50 kg. Therefore, rivaroxaban could be more easily administered and monitored in children requiring long-term anticoagulation than heparin or VKAs [26].

Despite a similar safety profile and an improved thrombotic burden linked to rivaroxaban therapy compared to standard anticoagulants, some pediatric patients might not benefit from rivaroxaban administration. This subset of patients was excluded from phase III trials due to an increased hemorrhage risk. Main exclusion criteria consisted of active bleeding or high risk of bleeding, eGFR < 30 mL/min/1.73 m^2^ (serum creatinine > 97.5th percentile in those aged < 1 year), hepatic disease with coagulopathy or alanine aminotransferase > 5 × ULN or total bilirubin > 2 × ULN (direct bilirubin > 20%), low platelet count, hypertension, and life expectancy < 3 moths. Additionally, children aged < 6 months were enrolled if they had bodyweight > 2600 g, oral feeding > 10 days, and gestational age at birth > 37 weeks. Thus, in these cases, rivaroxaban should be avoided unless new data will support the use of rivaroxaban in high-risk patients instead of classic antithrombotic strategies [26,32].

#### 3.4.3. Apixaban—Growing Evidence in Children

Most studies investigating the efficacy and safety of apixaban in children with VTE are ongoing, and the results are not currently available (Table 2). Only one study published the results on apixaban treatment in secondary prophylaxis of VTE in pediatric patients [27]. This study enrolled 15 patients aged 12–21 with bodyweight > 40 kg. Apixaban was initiated in the first 72 h from the VTE diagnosis, and the patients were followed up for 90 days. The dosing regimen was similar to adults, and apixaban 10 mg was administered twice a day during the first week, followed by the standard dose of 5 mg twice daily. Notably, 55% of patients experienced complete thrombosis resolution, while in the rest of the patients, the authors observed a reduction in thrombotic burden. No VTE recurrence was recorded during the follow-up period. Therefore, apixaban appeared efficient in VTE therapy in older children; however, the data should be confirmed in large clinical trials and younger children [27].

The SAXOPHONE study was a multicenter, randomized, open-label clinical trial designed to evaluate the utility of apixaban in the prevention of thromboembolic events in children with heart disease (congenital or acquired) as compared to standard anticoagulants (NCT02981472) [33]. Although study findings have not been published yet, the recruitment status is completed, and the authors reported some results on clinicaltrials.gov [34]. One hundred ninety-two participants aged ≤ 18 years had a prior thrombotic event. Patients with high bleeding risk and those with intracranial malformation or tumor, liver dysfunction, renal function < 30% of normal abilities, and thrombocytopenia were excluded from the study. The study’s main objective was to investigate apixaban safety in the pediatric population without primary efficacy outcomes [33]. The available data on clinicaltrials.gov showed that the authors reported a lower incidence of major or clinically relevant nonmajor bleeding events in the apixaban group compared to traditional anticoagulants (0.8% vs. 4.8%) during 12 months of follow-up. One patient from the apixaban group and one treated with LMWH or VKA experienced a major bleeding event. Notably, neither group reported thromboembolic events, and deaths were linked to thromboembolic events [34].

Authors from a phase III randomized, open-label, multicenter clinical trial reported results in the clinicaltrials.gov database, although they have not been published yet (NCT02369653) [35]. Children (n = 512) aged 1–18 years with acute lymphoblastic leukemia or lymphoma and a central venous device in whom chemotherapy was initiated enrolled in the study. The study’s main objective was to evaluate the potential benefit of apixaban in preventing thromboembolic events compared to no systemic prophylactic anticoagulation. The rate of VTE, cerebral venous sinus thrombosis, and VTE-related death was lower in the apixaban group (12.1% vs. 17.6% in patients without anticoagulation), with a similar rate of major bleedings (0.8% in both groups) [35].

Another phase IV randomized clinical trial investigating treatment with Apixaban in pediatric patients with VTE is expected to end in 2023 (NCT02464969) [36]. The primary outcomes will include safety endpoints (major and nonmajor bleedings), as well as efficacy endpoints (recurrent VTE and VTE-related mortality) [36].

#### 3.4.4. Edoxaban—A Valid Option in Children?

There are no existing published data on the safety and efficacy of edoxaban in children with VTE. Nevertheless, ongoing clinical trials aim to establish possible advantages of edoxaban before classic anticoagulant approaches (Table 2) [37]. One randomized, open-label, multicenter study enrolled 168 children with cardiac diseases at risk of thromboembolic events (NCT03395639). The authors will analyze safety and efficacy outcomes during a follow-up of 13 months. However, the results are not available and are awaiting for being published [37].

The edoxaban Hokusai VTE PEDIATRICS study was an open-label, randomized trial that evaluated the utility of edoxaban in pediatric VTE treatment. Results are expected to be published in 2022 (NCT02798471). Children aged < 18 years who received at least five days of parenteral anticoagulants (UFH, LMWH, or fondaparinux) were included in the study. Efficacy, safety, pharmacokinetic, and pharmacodynamic outcomes were considered for the analysis. Therefore, the results from these studies could provide a better understanding and a relatively solid background for edoxaban administration in children with VTE. Currently, the lack of data does not allow one to advocate in favor of edoxaban treatment in children.

## 4. Discussion

Summarizing recommendations from guidelines and key findings from analyzed clinical trials in pediatric patients with acute VTE heparin therapy should be initiated and continued for at least five days. Although OAT with VKAs could be instituted after heparin therapy, clinical studies highlighted that some DOACs were safe and efficient in pediatric VTE [13].

Dabigatran and rivaroxaban are the only two DOACs with extensively published data about children, and documented similar safety and efficacy profiles compared to standard therapy [21,22]. Moreover, the thrombosis resolution rate was improved in patients receiving rivaroxaban [5]. In the case of apixaban and edoxaban, results from ongoing clinical studies are awaited before introducing them into clinical practice. Consequently, bodyweight-adjusted doses of dabigatran and rivaroxaban might be considered in children diagnosed with VTE as alternatives to VKAs. Recommended dabigatran and rivaroxaban doses in children were provided in Table S1 [28] and Table S2 [30]. Nevertheless, DOACs should be avoided in children with high bleeding risk and those with severe renal dysfunction and hepatic impairment due to a lack of data in this subgroup of patients [21,22].

However, the results from clinical trials should be interpreted cautiously due to existing limitations. The majority of the analyzed studies were observational with a single-arm design. Only two published studies were randomized, controlled, and compared DOACs with standard anticoagulant therapies [5,21]. In addition, population samples were relatively low across studies; thus, extrapolating the results to “real-world” patients should be critically judged. Moreover, the efficacy of DOACs in clinical contexts, such as thrombophilia and antiphospholipid syndrome, was not established, as studies were underpowered [21].

Additionally, to the best of our knowledge, there are no existing data on DOACs’ utility as a stand-alone therapy in acute VTE without being preceded by heparin therapy. Thus, DOACs should be used after classic heparin therapy unless new data is available to shift the paradigm. Regarding primary prophylaxis, DOACs were studied in children who underwent the Fontan procedure to prevent thrombotic events, with similar efficacy and safety endpoints compared to aspirin [38]. However, primary prophylaxis data in VTE are lacking, and further research is required before making recommendations.

## 5. Conclusions

The incidence of thromboembolic events is relatively low in the general population, but it increases in hospitalized children and those who underwent thrombogenic procedures. The standard therapeutic approach in children with VTE is parenteral anticoagulation with UFH or LMWH. However, initiating OAT after 5–7 days of heparin therapy in VTE patients is reasonable. OAT is particularly interested in children requiring long-term antithrombotic therapy due to difficulties attributed to parenteral anticoagulation. VKAs could be used as an alternative to heparin even in the subacute phase of VTE. In addition to classic VKAs therapy, DOACs have been extensively studied in the last decade in pediatric patients. Clinical trials highlighted that rivaroxaban and dabigatran had predictable pharmacokinetic and pharmacodynamic profiles in children, similar to those in adults. Moreover, dabigatran and rivaroxaban had at least a similar safety profile to standard therapy but improved thrombotic burden and resolution during follow-up. Therefore, dabigatran and rivaroxaban could be valid therapeutic options for VTE management in children. In the case of apixaban and edoxaban, results from ongoing clinical studies are required before using them in pediatric VTE. Large, randomized clinical trials are needed to confirm the results. In addition, further research should investigate the utility of DOACs as a stand-alone therapy in acute VTE treatment, primary VTE prophylaxis, and particular contexts, such as antiphospholipid syndrome and thrombophilia.

## Figures and Tables

**Table 1:** Evidence from clinical trials regarding DOAC use in children with VTE.

Study, Year	Design	Patients	Age	Intervention	Comparator	Outcomes	Follow-Up
Halton et al., 2017 [19]	Multicenter, open-label, single-arm, phase IIa study	18 children who completed VTE treatment (UFH, LMWH, or OAT)	1–12 years	Dabigatran in oral liquid formulation (the equivalent of 150 mg in adults)	NA	(a) Dabigatran had similar and predictable PK and PD profiles compared to adults (b) No reported severe bleeding events, recurrent VTE, or other adverse events	30 days
Halton et al., 2017 [20]	Multicenter, open-label, single-arm, phase IIa study	8 children who completed VTE treatment (UFH, LMWH)	<12 months	Dabigatran in oral liquid formulation (the equivalent of 150 mg in adults)	NA	(a) Dabigatran had similar and predictable PK and PD profiles compared to adults (b) No reported bleeding events, death, or other adverse events	37 days
Halton et al., 2021 [21]	Multicenter, randomized, open-label, parallel-group, phase 2b/3 study(DIVERSITY)	328 children treated initially with UFH or LMWH	<18 years	Age- and weight-adjusted dose of Dabigatran	Standard anticoagulants (UFH, LMWH, VKAs, fondaparinux)	(a) Primary composite outcome (complete thrombus resolution, freedom from recurrent VTE, death) was similar in both groups (b) Risk of major bleeding events was similar (HR 0.94, 95% CI, 0.17–5.16, *p* = 0.95)	4 months
Brandao et al., 2020 [22]	Open-label, single-arm, prospective cohort study	203 children treated with standard therapy for ≥3 months or completed DIVERSITY study	3 months–18 years	Age- and weight-adjusted dose of Dabigatran	NA	(a) No reported deaths (b) 1% of patients had VTE recurrence (c) Major bleedings were reported in 1.5% of patients (d) Minor bleedings were observed in 18.2% of patients	12 months
Willman et al., 2018 [23]	Multicenter, phase I study (EINSTEIN-Jr)	59 children who completed VTE treatment	0.5–18 years	Bodyweight-adjusted single dose of rivaroxaban	NA	Plasma concentration-time profile was within 90% prediction interval, derived from PK modeling	NA
Kubitza et al., 2018 [24]	Multicenter, open-label, phase I study (EINSTEIN-Jr)	59 children who completed VTE treatment	0.5–18 years	Bodyweight-adjusted single dose of rivaroxaban	NA	(a) Rivaroxaban had predictable PK profile (b) Rivaroxaban was well tolerated, with no reported deaths, major or nonmajor bleeding events	23 days
Monagle et al., 2019 [25]	Multicenter, single-arm, phase II studies	93 children with VTE treated with LMWH or VKAs for at least 2 months (6 weeks for CR-VTE)	Birth to 17 years	Bodyweight-adjusted 20 mg-equivalent doses of rivaroxaban (once-daily, twice-daily, or three times daily)	NA	(a) No reported major bleeding events (b) 4% of patients had nonmajor bleeding events (c) No symptomatic recurrent VTE	30 days
Young et al., 2019 [26]	Multicenter, open-label, phase III study	335 children with VTE, treated initially with heparin or LMWH	Birth to 17 years	Bodyweight-adjusted dose of rivaroxaban (once-daily, twice-daily, or three times daily)	NA	(a) No reported major bleeding events (b) 3.2% of patients had nonmajor bleedings (c) Repeat imaging: 39.2% normalized, 39.6% improved, 0.3% deteriorated, no relevant changes in 5.1%	3 months
Male et al., 2019 [5]	Multicenter, randomized, open-label, phase III study	500 children with VTE were treated initially with heparin	Birth to 17 years	Bodyweight-adjusted 20 mg-equivalent doses of rivaroxaban	Standard anticoagulants (heparin or VKAs)	(a) Symptomatic recurrent VTE: HR 0.40, 95% CI, 0.11–1.41 (b) Major or nonmajor bleedings: HR 1.58, 95% CI, 0.51–6.27 (c) Only nonmajor bleedings in the rivaroxaban group (two major bleedings in heparin/VKAs group)	91 days
Pinchinat et al., 2019 [27]	An observational, single-arm pilot study	15 patients with bodyweight > 40 kg who experienced a primary VTE event	12–21 years	Apixaban initiated within 72 h of VTE diagnosis	NA	(a) Thrombus resolution in 55% of cases and a reduction in thrombotic burden in the rest of the patients (b) No recurrent VTE was reported	90

CR-VTE = catheter-related VTE; DOACs = direct oral anticoagulants; LMWH = low molecular weight heparin; OAT = oral anticoagulation therapy; PK = pharmacokinetic; VKA = vitamin K antagonist; VTE = venous thromboembolism.

**Table 2:** Ongoing clinical trials investigating antithrombotic therapy with DOAC in children.

Study	Design	Context	Intervention	Outcomes
NCT02981472	Multicenter, open-label, randomized study	Pediatric patients with congenital or acquired heart disease requiring chronic anticoagulation	Apixaban for thromboembolism prevention versus VKAs	(a) Major or clinically relevant nonmajor bleeding events (b) Incidence of thrombotic events and death related to thromboembolic events (c) All-cause death
NCT02369653	Multicenter, open-label, randomized study	Children with acute lymphoblastic leukemia or lymphoma treated with Asparaginase	Apixaban for thromboembolism prevention versus no anticoagulation	(a) Incidence of VTE, cerebral venous sinus thrombosis, and death related to VTE (b) Major bleeding (c) Clinically relevant nonmajor bleeding
NCT02464969	Open-label, randomized study	Children with VTE requiring anticoagulation	Apixaban versus standard of care (heparin, low molecular weight heparin, VKAs)	(a) Major and clinically relevant nonmajor bleeding events (b) Symptomatic and asymptomatic recurrent VTE and mortality linked to VTE
NCT03395639	Multicenter, open-label, randomized study	Children with cardiac disease with a high risk of thromboembolic events	Edoxaban versus standard of care (low molecular weight heparin, VKAs)	(a) Major and clinically relevant nonmajor bleeding events (b) Symptomatic thromboembolic events (c) Death related to thromboembolic events
NCT02798471	Multicenter, open-label, phase III, randomized study	Children with confirmed VTE	Edoxaban versus standard of care (low molecular weight heparin, heparin, VKAs, fondaparinux)	(a) Symptomatic recurrent VTE (b) Death linked to VTE (c) Thrombotic burden resolution/extension (d) Major bleeding (e) Clinically relevant nonmajor bleeding

DOACs = direct oral anticoagulants; VKAs = vitamin K antagonists; VTE = venous thromboembolism.

## Supplementary Materials

The following supporting information can be downloaded at: https://www.mdpi.com/article/10.3390/children9071093/s1, Table S1: Age- and bodyweight-adjusted doses of dabigatran (oral pellets) in children as recommended by the FDA [28]; Table S2: Bodyweight-adjusted doses of rivaroxaban in children as recommended by the FDA [30].
