# Supplementary material for: Current Antithrombotic Therapy Strategies in Children with a Focus on Off-Label Direct Oral Anticoagulants—A Narrative Review
Source: Children (Basel). 2022 Jul 21;9(7):1093. doi: 10.3390/children9071093 (PMC9319670; doi:10.3390/children9071093)
Supplement: Supplementary file 1 [file children-09-01093-s001.zip › children-1836159-supplementary.pdf]

Table S1. Age- and bodyweight-adjusted doses of dabigatran (oral pellets) in children as recommended by the FDA [28].

Children aged ≤ 2 years		
**Weight (kg)**	**Age (months)**	**Dose (mg) twice daily**
3 to < 4 kg	3 to < 6 months	30 mg
4 to < 5 kg	3 to < 10 months	40 mg
5 to < 7 kg	3 to < 5 months	40 mg
	5 to < 24 months	50 mg
7 to < 9 kg	3 to < 4 months	50 mg
	4 to < 9 months	60 mg
	9 to < 24 months	70 mg
9 to < 11 kg	5 to < 6 months	60 mg
	6 to < 11 months	80 mg
	11 to < 24 months	90 mg
11 to < 13 kg	8 to < 18 months	100 mg
	18 to < 24 months	110 mg
13 to < 16 kg	10 to < 11 months	100 mg
	11 to < 24 months	140 mg
16 to < 21 kg	12 to < 24 months	140 mg
21 to < 26 kg	18 to < 24 months	180 mg
Children between 2 years and < 12 years old		
7 to < 9 kg		70 mg
9 to < 11 kg		90 mg
11 to < 13 kg		110 mg
13 to < 16 kg		140 mg
16 to < 21 kg		170 mg
21 to < 41 kg		220 mg
≥ 41 kg		260 mg

Table S2. Bodyweight-adjusted doses of rivaroxaban in children as recommended by the FDA [30].

**Dosage Form**	**Weight (kg)**	**Dosage (mg)**			**Daily Dose**
		**Once a Day**	**Two Times a Day**	**Three Times A Day**	
Only oral suspension	2.6 to 2.9 kg			0.8 mg	2.4 mg
	3 to 3,9 kg			0.9 mg	2.7 mg
	4 to 4.9 kg			1.4 mg	4.2 mg
	5 to 6.9 kg			1.6 mg	4.8 mg
	7 to 7.9 kg			1.8 mg	5.4 mg
	8 to 8.9 kg			2.4 mg	7.2 mg
	9 to 9.9 kg			2.8 mg	8.4 mg
	10 to 11.9 kg			3 mg	9 mg
	12 to 29.9 kg		5 mg		10 mg
Oral suspension or tablets	30 to 49.9 kg	15 mg			15 mg
	≥ 50 kg	20 mg			20 mg
